# Bullying among medical students: prevalence, determinants, and implications for the educational environment

**DOI:** 10.3389/fmed.2026.1770390

**Published:** 2026-03-04

**Authors:** Shahd Al-Ghawi, Maryam Alwahaibi, Fatema Alajaimi, Mohammed Al-Badi, Hoor Alhabsi, Maria AL Azri, Sanjay Jaju, Nasar Alwahaibi

**Affiliations:** College of Medicine and Health Sciences, Sultan Qaboos University, Muscat, Oman

**Keywords:** bullying, healthcare professions education, learning environment, medical students, student wellbeing

## Abstract

**Background:**

Bullying in medical education negatively affects student wellbeing, learning engagement, and the educational climate. This study examined the prevalence, forms, impacts, and associated factors of bullying among medical students at a Middle Eastern university.

**Methods:**

A cross-sectional online survey was conducted among 288 medical students between October and December 2024 using a validated questionnaire capturing sociodemographic characteristics and bullying experiences. Descriptive statistics, chi-square tests with Benjamini–Hochberg correction, and binary logistic regression were applied.

**Results:**

Bullying prevalence was 11.5% (95% CI: 8.0–15.7). Classmates were the most frequently reported perpetrators (87.9%), and verbal bullying was the predominant form (90.9%), most commonly occurring in classroom settings. Appearance and personal characteristics were commonly cited perceived triggers. Reported consequences included disengagement from learning activities (42.4%) and low mood (30.3%). Logistic regression indicated a potential association between personal mobile data use and bullying exposure (OR = 0.387, 95% CI: 0.159–0.944, *p* = 0.04).

**Conclusion:**

Although prevalence was relatively modest, the findings highlight bullying as a concern for educational quality and student wellbeing. The study provides baseline evidence to inform institutional prevention strategies, reporting mechanisms, and support initiatives aimed at providing safer and more inclusive learning environments in medical education.

## Background

Bullying is defined as repeated and persistent aggressive behavior, whether direct or indirect, directed at an individual (1). It may occur in physical, verbal, emotional, social, or digital forms and has been documented across multiple educational and professional settings, including universities and online learning environments (2). Within medical education, bullying can manifest through verbal insults, social exclusion, rumor-spreading, or academically related behaviors such as excessive workload, intimidation, or misuse of authority, collectively described as academic bullying (3). These behaviors are consistently associated with adverse mental health outcomes, including stress, depression, burnout, sleep disturbances, and suicidal ideation, as well as reduced academic engagement, lowered motivation, and impaired professional development (4–10).

Although bullying has been widely investigated in school and workplace contexts, it remains comparatively underexplored in higher education, particularly within medical training environments (11). Medical students may be uniquely vulnerable due to hierarchical structures, high performance expectations, and the pressures of clinical and academic learning (12). Regional evidence from the Middle East and Gulf area indicates substantial prevalence. For example, a study in Ras Al Khaimah, UAE, reported a 34.1% prevalence among medical, nursing, dental, and pharmacy students (13). In Saudi Arabia, bullying among medical students has been reported with figures varying from 28 to 44% (14, 15). Similarly, 71.1% of medical students in Egypt reported bullying, most commonly verbal abuse (16). Despite these regional figures highlighting a significant concern within higher education, to our knowledge, there remains a notable gap in research focusing specifically on the prevalence and characteristics of bullying among medical students in Oman. This study aims to address this gap by providing initial evidence for Oman.

Despite growing global recognition of bullying as a behavioral determinant of health, research within the Middle East, and specifically in undergraduate medical education, remains limited. Important gaps persist regarding bullying related to academic specialization, social exclusion, and institutional climate (17). From a quality-improvement perspective, bullying represents a threat to the safety, inclusiveness, and psychological climate of the learning environment, key indicators of educational quality. To address these gaps, the present study examined the prevalence, forms, perceived effects, and associated factors of bullying among medical students. By applying a behavioral-health lens to understand this phenomenon, the study aims to generate evidence that can inform institutional policies, early-detection mechanisms, and targeted quality-improvement initiatives to promote safer and more supportive medical learning environments.

## Methods

### Ethical consideration

This study was conducted in accordance with the guidelines of the Declaration of Helsinki and received ethical approval from the Medical Research Ethics Committee (MREC), College of Medicine and Health Sciences, Sultan Qaboos University (SQU), Oman, with an approval number of MREC #3300. The cross-sectional observational study was carried out from October 2024 to December 2024. Prior to participation, all students received detailed information about the study purpose, confidentiality protections, and their rights as participants. Written informed consent was obtained from all participants. For participants aged 17 years, additional written informed consent was obtained from their legal guardians in accordance with guidelines for research involving minors. Participants aged 18 years and older provided their own independent written informed consent. Participation was entirely voluntary, and students were explicitly informed of their right to refuse or withdraw from the study at any stage without any consequences. Given the sensitive nature of some survey items, including those pertaining to self-harm and suicidal ideation, stringent ethical safeguards were implemented. All responses were collected anonymously to ensure participant privacy and encourage honest reporting. The online questionnaire platform was configured not to collect any identifiable information, ensuring complete anonymity. Researchers involved in data collection and handling were trained in ethical data management and participant well-being protocols to uphold these standards.

### Study design

This cross-sectional study was conducted at the College of Medicine and Health Sciences at SQU. The inclusion criteria encompassed all Omani MD students enrolled at the institution at any age. To ensure comprehensive validation of the instrument, questionnaire items underwent rigorous review by a panel of experts in medical education, psychology, and public health for content validity and context-specific construct validity. This process involved assessing whether adapted items from validated bullying measures (18, 19) accurately captured the nuanced forms of bullying prevalent in medical training environments, differentiating them from general youth bullying experiences. These items were carefully revised and supplemented with self-developed items to reflect specific academic pressures, hierarchical dynamics, and clinical interactions unique to medical education. The expert panel refined item wording to ensure relevance, interpretability, and cultural appropriateness within the Omani medical student context.

For translation, the survey was initially developed in English and then independently translated into Arabic by two professional native Arabic-speaking translators proficient in medical terminology. A third native speaker then performed a back-translation to English, and any discrepancies were resolved through discussion to ensure semantic and conceptual equivalence.

While the conceptual definition of bullying adopted for this study [repeated and persistent aggressive behavior, whether direct or indirect, directed at an individual (1)] was used as the theoretical basis for questionnaire development, participants were not explicitly provided with this definition at the outset of the survey. Instead, their understanding of bullying was implicitly guided by a comprehensive range of specific bullying-related questions presented within the questionnaire. For instance, the primary outcome, ‘ever experienced bullying at SQU during your study,’ was assessed via a single direct question: ‘Have you ever experienced bullying at SQU during your study? (Yes/No).’ This approach, while providing a broad screening measure, was chosen as it is commonly employed in large-scale prevalence studies for initial identification. We acknowledge that a single-item measure may under-detect bullying due to varied subjective interpretations of ‘bullying’ or reluctance to label experiences as such. For those who responded ‘Yes’ to this screening question, subsequent detailed questions probed specific forms (e.g., ‘What type of bullying have you faced?’) and perpetrators (e.g., ‘Who has bullied you?’), drawing from both self-developed items and validated measures from the aforementioned validated measures.

A pilot study with 15 MD students (excluded from analysis) assessed the clarity and internal reliability of the instrument. Cronbach’s alpha for six bullying-related items (e.g., Have you ever been bullied at school? Have you witnessed bullying at SQU? Have you ever bullied other students?) was *α* = 0.75, indicating acceptable internal consistency. Test–retest reliability was not conducted for this cross-sectional study.

### Participants

SQU admits 130 medical students annually (65 males and 65 females) into a six-year medical program. The curriculum comprises three phases: basic sciences, integrated system-based learning with early clinical exposure, and clinical rotations across major specialties. To enhance accuracy and data quality, questionnaire clarity was validated, detailed instructions were provided, and confidentiality assurances were emphasized to promote honest responses. The calculated target sample size was 288 students. From an initial pool of 322 survey submissions, 306 provided informed consent. To ensure a complete dataset for analysis, 18 incomplete or inconsistent responses were removed via listwise deletion. This process yielded a final analytic sample of 288 participants with no missing data, thereby enhancing sampling transparency.

### Sample size calculation

The required sample size was calculated using the formula *n* = NZ^2^p(1–p)/{d^2^(N–1) + Z^2^p(1–p)}, where *N* = 780 enrolled MD students, Z = 1.96 (95% confidence), d = 5% margin of error, and *p* = 50% estimated proportion. To account for potential incomplete responses, the target was increased by 10%, resulting in a final sample size of 288 students, which was achieved. This represents approximately one-third of the student body.

### Data collection

The questionnaire consisted of four sections. The first provided study information, including objectives, procedures, and ethical approval. The second obtained informed consent. The third collected sociodemographic data (gender, age, marital status, year of study, parental education, family economic status, self-rated health, and internet access). The final section assessed bullying experiences, including prevalence, frequency, perpetrators, types of bullying, and perceived effects. Full survey items are provided in Supplementary material 1 to support transparency and reproducibility. Most items were closed-ended (yes/no or single-choice) to enable quantitative analysis and consistent response interpretation. The instrument was aligned with validated higher education bullying tools, with adaptations to the local context.

### Data analysis

Data were analyzed using IBM SPSS Statistics version 29.0 (20). Descriptive statistics were used to summarize categorical variables as frequencies and percentages. Associations between independent variables and bullying were assessed using the Chi-square test; when expected counts in crosstabs fell below assumptions, Fisher’s exact test was applied. To account for multiple comparisons, the Benjamini–Hochberg (BH) procedure was applied across the set of univariate tests to control the false discovery rate (FDR) at 25% (Q = 0.25). BH critical thresholds were computed as (i/m) × Q, where i is the rank of the raw *p*-value and m is the total number of tests. Variables with BH critical threshold < 0.10 were selected *a priori* for inclusion in the multivariable binary logistic regression model. A two-sided *p*-value <0.05 after correction was considered statistically significant.

## Results

This study included 288 participants, with the majority (53.8%) aged between 20 and 22 years. Females dominated the sample (60.1%). More than half of the students (50.3%) lived with their families, while 35.8% lived in shared accommodations, and 13.9% lived alone. Nearly all students (99.0%) were single. Regarding study phases, 31.2% were in Phase 1, 41.0% in Phase 2, and 27.8% in Phase 3. The majority (78.1%) were from outside the Muscat city. Regarding parental education, 37.2% of fathers had an undergraduate degree, while 27.8% had a postgraduate degree, and 44.8% of mothers had an undergraduate, and 11.1% had a postgraduate education. Financially, more than half (54.2%) belonged to high-income families, 38.9% were from middle-income families, and the remaining 11.1% were from low-income families. The student’s health was reported good by most of them (64.2%), 33.0% reported excellent health status, and 2.8% were below average health. The majority of students (98.3%) were frequent internet users, with 92.0% access the internet through Wi-Fi at SQU, 89.6% access internet at home, 85.4% using personal mobile data, and 28.1% using various Wi-fi hotspots (Table 1).

**Table 1:** Sociodemographic characteristics of medical students at Sultan Qaboos University, Oman.

Variables	n (288)	%
Age in years (*n* = 288)		
17–19	86	29.9
20–22	155	53.8
23–25	47	16.3
Gender (*n* = 288)		
Male	115	39.9
Female	173	60.1
Where do you live? (*n* = 288)		
Alone	40	13.9
Shared living (Dorms/Roommates/Flat)	103	35.8
With your own family	145	50.3
Marital status (*n* = 288)		
Single	285	99
Previously / currently married	3	1
Year of study (*n* = 288)		
Phase 1	90	31.2
Phase 2	118	41
Phase 3	80	27.8
Place of original residence (*n* = 288)		
Muscat	63	21.9
Outside capital	225	78.1
Father’s educational level (*n* = 288)		
Pre-university education	101	35.1
Under graduate	107	37.2
Postgraduate	80	27.8
Mother’s educational level (*n* = 288)		
Pre-university education	127	44.1
Under graduate	129	44.8
Postgraduate	32	11.1
Family economic status (*n* = 288)		
Low: monthly income less than 500 OMR	20	6.9
Middle: monthly income between 500 and 1,500 OMR	112	38.9
High: monthly income more than 1,500 OMR	156	54.2
How would you rate your general health? (*n* = 288)		
Below Average	8	2.8
Good	185	64.2
Excellent	95	33
How often do you use the internet? (*n* = 288)		
Infrequent users	5	1.7
Frequent users	283	98.3
What methods do you usually use to access the internet?		
Wi-Fi connection at SQU campus	265	92
Wi-Fi connection at Home	258	89.6
Personal mobile data	246	85.4
Wi-Fi hotspots	81	28.1

SQU, Sultan Qaboos University.

Around 43.8% of students reported being bullied in schools, while 25.3% experienced bullying at home. The prevalence of bullying was 11.5% (95% CI: 8.0–15.7). Among the total sample, around 31.9% had witnessed bullying somewhere in SQU, and 13.5% admitted to bullying others, mostly (76.9%) through verbal bullying, and through friends (43.6%). The main reported reasons for bullying were ‘fun’ (74.4%) and personal issues (23.1%). The majority (84.7%) of students reported that SQU should have a law to protect students from bullying (Table 2).

**Table 2:** Distribution of bullying among medical students at Sultan Qaboos University, Oman.

Variables	n (288)	%
Have you ever been bullied at school? (*n* = 288)		
Yes	126	43.8
No	162	56.3
Have you ever been bullied at home? (*n* = 288)		
Yes	73	25.3
No	215	74.7
Have you ever experienced bullying at SQU during your study? (*n* = 288)		
Yes	33	11.5
No	255	88.5
Have you witnessed bullying at SQU? (*n* = 288)		
Yes	92	31.9
No	196	68.1
If yes, what was your reaction? (*n* = 92)		
Tried to interfere	34	37
Not reacted in any way	58	63
Have you ever bullied other students? (*n* = 288)		
Yes	39	13.5
No	249	86.5
Have you used any of the following to bully other students? (*n* = 39)		
Online video clips of them	1	2.6
Online video clips of you	3	7.7
Chatroom	8	20.5
Through friends	17	43.6
Verbal bullying	30	76.9
Picture messages	4	10.3
What makes you bully other students? (*n* = 39)		
Lack of awareness	2	5.1
They provoke me	2	5.1
Due to personal issues	9	23.1
For fun	29	74.4
To feel more powerful	4	10.3
Do you think there should be a law at SQU to protect students from bullying? (*n* = 288)		
Yes	244	84.7
No	44	15.3

SQU, Sultan Qaboos University.

Of the 33 students reported to be bullied at SQU, almost half (45.5%) experienced it a few times during academic term and 27.3% faced it once a year. Most (87.9%) bullying was committed by classmates, followed by teachers/instructors (30.3%). The most common type of bullying was verbal (90.9%), followed by mental/emotional (42.4%). The perceived effects of bullying were negative, with 42.4% reported disengagement, 30.3% felt low, and 27.3% experienced low self-esteem and lack of motivation. The method of bullying mostly occurred through verbal bullying (75.8%), most of them happened in classrooms (66.7%), resting rooms (39.4%), and corridors (39.4%). The most common reasons for being bullied were appearance and personal traits, which accounted for 69.7, and 60.6%, respectively. Among the bullied students, majority of them (84.8%) did not complain about the incidents. The main reason for not complaining was “not important” (60.6%) and felt it will stop by itself 21.2% (Table 3).

**Table 3:** Features of bullied medical students at Sultan Qaboos University, Oman.

Variables	n (33)	%
How often do you get bullied at SQU? (*n* = 33)		
Daily	3	9.1
Weekly	3	9.1
Monthly	3	9.1
Once a year	9	27.3
A few times during the academic term	15	45.5
Who has bullied you? (*n* = 33)		
Roommates	7	21.2
Classmates	29	87.9
Hospital staff	7	21.2
Patients	4	12.1
Administrative staff	4	12.1
Teachers/ instructors	10	30.3
What type of bullying have you faced? (*n* = 33)		
Verbal	30	90.9
Physical	1	3.0
Cyber	3	9.1
Mental or emotional	14	42.4
What are the negative effects of bullying on you? (*n* = 33)		
Drug misuse	1	3.0
Self-harm	1	3.0
Suicidal Thoughts	2	6.1
Social anxiety	6	18.2
Change in appetite	5	15.2
Disengagement	14	42.4
Lack of motivation	9	27.3
Feeling low	10	30.3
Difficulty concentrating in class	8	24.2
Depression	7	21.2
Poor marks	4	12.1
Low self-esteem	9	27.3
Hate and anger	8	24.2
Fearfulness	6	18.2
Nothing	5	15.2
Which of the following methods have been used to bully you? (*n* = 33)		
Online video clips of them	−1	−1
Online video clips of you	2	6.1
Chatroom	6	18.2
Through friends	11	33.3
Verbal bullying	25	75.8
Picture messages	1	3.0
Where does bullying take place? (*n* = 33)		
SQU housing	1	3.0
Hospital	6	18.2
Resting rooms	13	39.4
Other transport	6	18.2
Cars	6	18.2
In the buses	6	18.2
Corridors	13	39.4
Classrooms	22	66.7
Cafeteria/coffee	12	36.4
Laboratory sessions	3	9.1
What do think makes them bully you? (*n* = 33)		
Joking	1	3.0
Academic excellence (e.g., discrimination due to your high grades)	5	15.2
Poor academic performance (e.g., low GPA, failing, or academic delay)	5	15.2
Exclusion from activities (e.g., being left out of events or social activities)	4	12.1
Due to your academic major (e.g., belittling your field of study)	5	15.2
Appearance or personal traits (e.g., physical appearance or manner of speaking)	23	69.7
Personal differences (e.g., way of thinking or opinions)	20	60.6
Have you complained about bullying?		
Yes	5	15.2
No	28	84.8
If no, reasons for not complaining		
Not important	20	60.6
Hope it will stop itself	7	21.2
Not sure to whom to complain	3	9.1
Afraid	2	6.1
Threatened not to complain	1	3.0

SQU, Sultan Qaboos University.

Older age students were more prevalent (30.3%) among bullied students compared with non-bullied students (14.5%), which was statistically significant in univariate analysis (*p* = 0.03). Phase 3 students were also more prevalent among bullied students (45.5% vs. 25.5%; *p* = 0.04). Use of personal mobile data was associated with bullying in univariate testing (*p* = 0.04). No other variables reached statistical significance in univariate analyses. BH critical thresholds were calculated and raw *p*-values were compared to those thresholds to identify BH-significant variables. Applying our prespecified rule (BH critical threshold < 0.10) identified the following variables for model entry: age, year of study, use of personal mobile data, living arrangement, and use of Wi-Fi hotspots (Table 4). These variables were entered into the binary logistic regression model.

**Table 4:** Comparison between bullied and non-bullied medical students at Sultan Qaboos University, Oman.

Variables	Have you ever experienced bullying at SQU during your study?		Raw *p*-value*	BH critical threshold
	Yes (*n* = 33) n (%)	No (*n* = 255) n (%)		
Age (*n* = 288)				
17–19	5 (15.2)	81 (31.8)	0.03*	0.017
20–22	18 (54.5)	137 (53.7)		
23–25	10 (30.3)	37 (14.5)		
Gender (*n* = 288)				
Male	15 (45.5)	100 (39.2)	0.49*	0.200
Female	18 (54.5)	155 (60.8)		
Where do you live? (*n* = 288)				
Alone	7 (21.2)	33 (12.9)	0.13*	0.083
Shared Living (Dorms/Roommates/Flat)	7 (21.2)	96 (37.6)		
With your own family	19 (57.6)	126 (49.4)		
Year of study (*n* = 288)				
Phase 1	6 (18.2)	84 (32.9)	0.04*	0.067
Phase 2	12 (36.4)	106 (41.6)		
Phase 3	15 (45.5)	65 (25.5)		
Place of original residence (*n* = 288)				
Muscat	9 (27.3)	54 (21.2)	0.43*	0.133
Outside capital	24 (72.7)	201 (78.8)		
Father’s educational level (*n* = 288)				
Pre-University Education	9 (27.3)	92 (36.1)	0.60*	0.217
Under graduate	14 (42.4)	93 (36.5)		
Postgraduate	10 (30.3)	70 (27.5)		
Mother’s educational level (*n* = 288)				
Pre-University Education	15 (45.5)	112 (43.9)	0.93*	0.250
Under graduate	15 (45.5)	114 (44.7)		
Postgraduate	3 (9.1)	29 (11.4)		
Family economic status–monthly income in OMR (*n* = 288)				
Low (<500)	2 (6.1)	18 (7.1)	0.49*	0.183
Middle (500–1,500)	16 (48.5)	96 (37.6)		
High (>1,500)	15 (45.5)	141 (55.3)		
How would you rate your general health? (*n* = 288)				
Below Average	2 (6.1)	6 (2.4)	0.47*	0.167
Good	21 (63.6)	164 (64.3)		
Excellent	10 (30.3)	85 (33.3)		
How often do you use the internet? (*n* = 288)				
Infrequent users	1 (3.0)	4 (1.6)	0.46^	0.150
Frequent users	32 (97.0)	251 (98.4)		
Using Wi-Fi connection at SQU campus (*n* = 288)				
Yes	30 (90.9)	235 (92.2)	0.74^	0.233
No	3 (9.1)	20 (7.8)		
Using Wi-Fi connection at Home (*n* = 288)				
Yes	28 (84.8)	230 (90.2)	0.36^	0.117
No	5 (15.2)	25 (9.8)		
Using personal mobile data (*n* = 288)				
Yes	24 (72.7)	222 (87.1)	0.04^	0.036
No	9 (27.3)	33 (12.9)		
Using Wi-Fi hotspots (*n* = 288)				
Yes	6 (18.2)	75 (29.4)	0.11*	0.071
No	27 (81.8)	180 (70.6)		

*Pearson Chi-square test, ^ Fisher’s exact test used when >20% of expected cell counts <5, Q = 0.25. Variables with BH critical threshold < 0.10 were selected for multivariable model. SQU: Sultan Qaboos University.

Binary logistic regression analysis showed that students who used personal mobile data had lower odds of reporting bullying compared with those who did not use personal mobile data (OR = 0.387, 95% CI: 0.159–0.944, *p* = 0.04). The association was statistically significant. Other factors, including age groups (20–22: *p* = 0.39; 23–25: *p* = 0.31), living arrangement (Shared Living: *p* = 0.21; With own family: *p* = 0.63), year of study (Phase 2: *p* = 0.68; Phase 3: *p* = 0.93), and using Wi-Fi hotspots (*p* = 0.43), were not statistically significant predictors of bullying in the multivariable model (Table 5).

**Table 5:** Logistic regression results for factors affecting bullied medical students at Sultan Qaboos University, Oman.

Variables	B coefficient	*p-*value	Odds	95% C. I. for odds	
				Lower	Upper
Age groups (Reference: 17–19)					
20–22	−0.754	0.39	0.470	0.084	2.618
23–25	−1.063	0.31	0.345	0.044	2.728
Where do you live? (Reference: Alone)					
Shared Living (Dorms/Roommates/Flat)	0.747	0.21	2.110	0.650	6.844
With your own family	0.246	0.63	1.279	0.468	3.496
Year of study (Reference: Phase 1)					
Phase 2	0.351	0.68	1.420	0.271	7.429
Phase 3	−0.087	0.93	0.916	0.147	5.713
Using personal mobile data (Reference: No)					
Yes	−0.949	0.04	0.387	0.159	0.944
Using Wi-Fi hotspots (Reference: No)					
Yes	0.390	0.43	1.477	0.563	3.877

CI, confidence interval.

## Discussion

This study provides new evidence on bullying among medical students, identifying a prevalence of 11.5%. Although lower than rates reported in several international settings, this still represents a meaningful concern for learner wellbeing and the quality of the educational environment (21–23). Moderate prevalence has been documented in the UAE (34.1%) (13), Tanzania (34.7%) (24), and Saudi Arabia (28–44%) (14, 15), while substantially higher figures have been reported in France (41.7%) (25), China (51.4%) (26), Ghana (81%) (27), and the United States (85%) (28). Such variation may reflect differences in measurement tools, reporting norms, academic culture, and willingness to disclose. The comparatively lower rate observed in this study may indicate that aspects of the learning environment are supportive; however, cultural reluctance to report bullying, concerns about academic repercussions, and the use of a single screening question may contribute to underestimation (29). From a quality-improvement perspective, underreporting is itself an indicator of a system-level vulnerability.

The use of a single screening question (‘Have you ever experienced bullying at SQU during your study?’) to determine the prevalence of bullying, while providing a direct measure, warrants discussion regarding potential misclassification. Such single-item measures are common in large-scale prevalence studies for initial identification, offering a broad overview. However, they rely heavily on participants’ subjective interpretation of ‘bullying,’ which can vary due to individual experiences, cultural norms, or awareness of what constitutes bullying. This approach may lead to both underestimation (e.g., if students do not label certain negative experiences as ‘bullying’ or fear repercussions for reporting) and, less commonly, overestimation (e.g., misinterpreting isolated incidents as bullying).

Verbal bullying emerged as the most common form, consistent with findings from Ghana (94.1%) (27), Turkey (92.6%) (30), Saudi Arabia (90%) (15), and Iran (85.5%) (31). Verbal bullying in medical training is often tied to hierarchical relationships, competitive academic pressures, and communication norms that may unintentionally normalize disrespectful behavior (23). Emotional bullying was also notable (42.4%), reflecting the psychological demands and performance expectations typical of medical education. While access to mental health services may buffer some effects, global evidence consistently demonstrates the significant psychological consequences of bullying for medical learners (7). These findings underline the importance of monitoring educational climate as part of institutional quality and safety frameworks.

Physical bullying was rare (3%), aligning with low rates in Saudi Arabia (4%) (15) and Turkey (5.5%) (30), but substantially lower than those reported in Lebanon (23.9%) (31), Iran (24.9%) (32), Jordan (32%) (33), and Bangladesh (82.2%) (34). Cyberbullying (9.1%) occurred less frequently than in India (38.5%) (35), Saudi Arabia (49.1%) (36), and Malaysia (24.4%) (37), and is comparable to UAE findings (4.7%) (38). These differences may reflect variations in institutional digital policies, levels of online monitoring, and student digital behavior norms. Such data are valuable for shaping targeted quality-improvement interventions, particularly around online professionalism and digital conduct.

Classmates were the most frequently reported perpetrators (87.9%), consistent with UAE findings (38), however, studies from Pakistan show bullying by consultants (39), and others have documented bullying from peers, supervisors, and clinical staff (28, 40, 41). This highlights the complex social ecology of bullying, where peer interactions, institutional culture, and academic stressors intersect. Most bullying occurred in classroom settings and was frequently related to appearance or differences in opinion, indicating the influence of social pressures alongside academic demands.

The perceived consequences, learning disengagement, low mood, diminished self-esteem, and reduced motivation, align with global evidence linking bullying to adverse educational and psychological outcomes (42). These outcomes have particular implications for medical education, where learner wellbeing is closely connected to clinical performance and, ultimately, patient safety. Binary logistic regression analysis indicated that students who used personal mobile data had lower odds of reporting bullying (OR = 0.387, 95% CI: 0.159–0.944, *p* = 0.04). As a cross-sectional study, this finding indicates an association, not a causal relationship, and could be influenced by various unmeasured confounding factors. It serves primarily as a hypothesis-generating observation that warrants further investigation with longitudinal designs to explore potential temporal dynamics or causal pathways.

Witnessing bullying was common (31.9%), with most bystanders not intervening (63%). This aligns with evidence from the UAE (22.8%) (38) and the United States (24.7%) (43). Bystander inaction may reflect uncertainty about reporting pathways, fear of retaliation, or a normalized culture of bullying. Furthermore, 13.5% of students reported bullying others, mainly through verbal behaviors (76.9%), a higher rate than that observed in the UAE (3.6%) (38). These findings suggest that students may occupy multiple roles, victim, perpetrator, and witness, highlighting the importance of institution-wide strategies to create a culture of psychological safety and shared responsibility.

No significant gender differences were found, contrasting with studies showing higher bullying rates among female students (44–46). Gender norms, cultural expectations, and sample characteristics may explain this discrepancy. Underreporting was apparent, consistent with global patterns in medical education. The comparatively lower prevalence observed in our study, while potentially indicating a more supportive academic environment, must also be interpreted in light of this challenge. Students often fear negative academic consequences, such as adverse evaluations or professional reprisal, or perceive reporting as futile if mechanisms are unclear or have not yielded positive outcomes in the past (47). Further, cultural reluctance to openly confront issues, particularly within hierarchical educational structures common in medical training, can contribute significantly to underestimation (22). Some students may also normalize bullying, viewing it as an inevitable ‘rite of passage’ rather than reportable bullying, which further masks the true prevalence (48). This lack of transparent, confidential, and effective reporting pathways creates a system-level vulnerability, indicating that students may not feel safe or empowered to disclose negative experiences. These findings underscore the urgent need for universities to establish and promote clear, trustworthy reporting systems, alongside institutional protections against retaliation and comprehensive education on recognizing and responding to bullying (47).

### Strengths and limitations

This study addresses an important, under-researched issue of bullying in medical education, providing novel data from a Middle Eastern setting with a high response rate (288/780 students). Methodological strengths include the use of both English and Arabic questionnaires for accessibility and cultural relevance, and adherence to an Institutional Review Board-approved protocol. The instrument’s inclusion of items on witnessing and perpetrating bullying, beyond just victimization, offers a broader view of the learning environment. Rigorous application of the Benjamini–Hochberg multiple-comparison correction and reporting of confidence intervals enhance the robustness of descriptive results. Despite these strengths, limitations include the cross-sectional design, which precludes establishing causal relationships, rendering findings hypothesis-generating. Reliance on self-report data is susceptible to recall and social desirability biases, and fear of disclosure, particularly in a conservative cultural context, may lead to underestimation of bullying prevalence (29). A convenience sample and single-institution scope limit external validity and generalizability to other medical schools. The brief, single-item bullying screening question may miss nuanced experiences and provides limited information on frequency or severity. Lastly, low bullying prevalence (33 events) constrained statistical power for subgroup analyses and contributed to potential logistic regression model instability. Future research should employ multi-item instruments and longitudinal designs to better capture bullying complexity and establish causal links.

## Conclusion

Bullying remains a persistent concern in medical education, even where overall prevalence is lower than international estimates. Verbal bullying predominated and was largely perpetrated by classmates in academic settings, contributing to disengagement, emotional distress, and reduced motivation. Underreporting emerged as a key challenge. While our cross-sectional design provides valuable prevalence data and identifies associations, it is important to note that these findings are hypothesis-generating and do not establish causal relationships. To promote safe, respectful, and inclusive learning environments, medical schools should consider these insights to develop comprehensive, system-level strategies, including clear reporting systems, professionalism training, wellbeing support, and ongoing monitoring. Future research employing longitudinal and multi-item validated instruments is recommended to establish causality and further delineate the complex dynamics of bullying in medical education.

## Data Availability

The original contributions presented in the study are included in the article/Supplementary material, further inquiries can be directed to the corresponding author.
