# Supplementary material for: Bullying among medical students: prevalence, determinants, and implications for the educational environment
Source: Front Med (Lausanne). 2026 Mar 4;13:1770390. doi: 10.3389/fmed.2026.1770390 (PMC12995646; doi:10.3389/fmed.2026.1770390)
Supplement: Supplementary file 1 [file Data_Sheet_1.pdf]

Section 1: Demographics

**1.**	**Age (in years):**<br>○ 17 – 19.<br>○ 20 – 22.<br>○ 23 – 25.<br>○ Above 25.
**2.**	**Gender:**<br>○ Male<br>○ Female
**3.**	**Where do you live?**<br>○ Alone<br>○ Shared Living (Dorms/Roommates/Flat)<br>○ With your own family
**4.**	**Marital status:**<br>○ Single.<br>○ Previously / Currently married
**5.**	**Year of study:**<br>○ Foundation<br>○ First<br>○ Second<br>○ Third<br>○ Fourth<br>○ Fifth<br>○ Sixth<br>○ Seventh<br>○ Other:………………………………………
**6.**	**Place of original residence:**<br>○ Muscat.<br>○ Dhofar.<br>○ Musandam.<br>○ Buraymi.<br>○ The Dakhiliyah.<br>○ The North Batinah.<br>○ The South Batinah.<br>○ The South Sharqiyah.<br>○ The North Sharqiyah.<br>○ The Dhahirah.<br>○ The Wusta.<br>Other: ………………………………..
**7.**	**Father's education level**<br>○ Illiterate<br>○ Primary school

	○ Secondary school<br>○ Diploma<br>○ Bachelor degree<br>○ Master<br>○ PhD<br>○ Others
**8.**	**Mother's educational level**<br>○ Illiterate<br>○ Primary school<br>○ Secondary school<br>○ Diploma<br>○ Bachelor degree<br>○ Master<br>○ PhD<br>○ Others
**9.**	**Family economic status (according to the National Centre for Statistics and Information, Oman, 2023)**<br>○ Low: monthly income less than 500 OMR<br>○ Middle: monthly income between 500 and 1500 OMR<br>○ High: monthly income more than 1500 OMR
**10.**	**How would you rate your health?**<br>○ Below Average<br>○ Good<br>○ Excellent
**11.**	**How often do you use the internet?**<br>○ Infrequent users<br>○ Frequent users
**12.**	**What methods do you usually use to access the internet?**<br>○ Wi-Fi connection at SQU campus<br>○ Wi-Fi connection at Home<br>○ Personal mobile data<br>○ Wi-Fi hotspots

Section II: General Bullying

Have you ever been bullied at school?

- Yes
- No

Have you ever been bullied at home?

- Yes
- No

Have you ever experienced bullying at SQU during your study?

- Yes
- No

Have you witnessed bullying at SQU?

- Yes
- No

If yes, what was your reaction?

- Tried to interfere
- Not reacted in any way

Have you ever bullied other students?

- Yes
- No

Have you used any of the following to bully other students?

- Online video clips of them
- Online video clips of you
- Chatroom
- Through friends
- Face to face messaging
- Picture messages

What makes you bully other students?

- Lack of awareness
- They provoke me
- Due to personal issues

- For fun
- To feel more powerful

Do you think there should be a law at SQU to protect students from bullying?

- Yes
- No

Features

How often do you get bullied at SQU?

- Daily
- Weekly
- Monthly
- Once a year
- A few times during the academic term

Who has bullied you?

- Roommates
- SQU mates
- Hospital staff
- Patients
- Administrative staff
- Teachers/ instructors

What type of bullying have you faced?

- Verbal
- Physical
- Cyber
- Mental or emotional
- Sexual

What are the negative effects of bullying on you?

- Drug misuse
- Self-harm
- Suicidal Thoughts
- Social anxiety
- Change in appetite

- Disengagement
- Lack of motivation
- Feeling low
- Difficulty concentrating in class
- Depression
- Poor marks
- Low self-esteem
- Hate and anger
- Fearfulness
- Nothing

Which of the following methods have been used to bully you?

- Online video clips of them
- Online video clips of you
- Chatroom
- Through friends
- Face to face messaging
- Picture messages

Where does bullying take place?

- SQU housing
- Hospital
- Resting rooms
- Other transport
- Cars
- In the buses
- Corridors
- Classrooms
- Cafeteria/coffee
- Laboratory sessions

What do think makes them bully you?

- Joking
- Academic excellence (e.g., discrimination due to your high grades)
- Poor academic performance (e.g., low GPA, failing, or academic delay)
- Due to your academic major (e.g., belittling your field of study)
- Exclusion from activities (e.g., being left out of events or social activities)
- Appearance or personal traits (e.g., physical appearance or manner of speaking)
- Personal differences (e.g., way of thinking or opinions)

Have you complained about bullying?

- Yes
- No

If no, reasons for not complaining

- Not sure to whom to complain
- Afraid
- Threatened not to complain
- Hope it will stop itself
- Not important
